# Seasonal and Geographic Dynamics in Bioproperties and Phytochemical Profile of *Limonium algarvense* Erben

**DOI:** 10.3390/molecules29020481

**Published:** 2024-01-18

**Authors:** Catarina Guerreiro Pereira, Maria João Rodrigues, Izabela Nawrot-Hadzik, Adam Matkowski, Luísa Custódio

**Affiliations:** 1 Centre of Marine Sciences (CCMAR/CIMAR LA), Campus of Gambelas, University of Algarve, 8005-139 Faro, Portugal; cagpereira@ualg.pt; 2 Department of Pharmaceutical Biology and Botany, Wroclaw Medical University, 50-367 Wroclaw, Poland; izabela.nawrot-hadzik@umw.edu.pl (I.N.-H.); pharmaceutical.biology@wp.eu (A.M.)

**Keywords:** halophyte plants, salt tolerant plants, sea lavender, Iberian endemism, antioxidant, anti-inflammatory, phenolics

## Abstract

This study delved into the influence of ecological and seasonal dynamics on the synthesis of secondary metabolites in the medicinal halophyte *Limonium algarvense* Erben, commonly known as sea lavender, and examined their antioxidant and anti-inflammatory properties. Aerial parts of sea lavender were systematically collected across winter, spring, summer, and autumn seasons from distinct geographic locations in southern Portugal, specifically “Ria de Alvor” in Portimão and “Ria Formosa” in Tavira. The investigation involved determining the total polyphenolic profile through spectrophotometric methods, establishing the chemical profile via liquid chromatography electrospray ionization quadrupole time-of-flight mass spectrometry (LC-ESI-QTOF-MS/MS), and evaluating in vitro antioxidant properties using radical and metal-based methods, along with assessing anti-inflammatory capacity through a cell model. Results unveiled varying polyphenol levels and profiles across seasons, with spring and autumn samples exhibiting the highest content, accompanied by the most notable antioxidant and anti-inflammatory capacities. Geographic location emerged as an influential factor, particularly distinguishing plants from “Ria de Alvor”. Seasonal fluctuations were associated with environmental factors, including temperature, which, when excessively high, can impair plant metabolism, but also with the presence of flowers and seeds in spring and autumn samples, which also seems to contribute to elevated polyphenol levels and enhanced bioproperties of these samples. Additionally, genetic factors may be related to differences observed between ecotypes (geographical location). This study underscores sea lavender’s potential as a natural source of antioxidant and anti-inflammatory agents, emphasizing the significance of considering both geographic location and seasonal dynamics in the assessment of phenolic composition and bioactive properties in medicinal plant species.

## 1. Introduction

Sea lavenders are a group of halophytic plants belonging to the genus *Limonium* Mill. (family Plumbaginaceae), including over 400 species thriving in coastal and saline environments worldwide [1]. *Limonium* plants are mainly perennial or annual herbs, and their leaves are disposed in rosettes at the base, where distinctive panicle-shaped inflorescences arise [1,2]. *Limonium* comprises many ornamental species valued for their vibrant calyces that persist even after the flowers fade, giving them their common name “statice,” but they also have a long ethnomedicinal tradition in China, Europe, Latin America, and Arabia for the treatment of various cardiovascular and inflammatory problems [3,4]. Moreover, studies have scientifically proven their medicinal value, such as anti-inflammatory, antibacterial, antiviral, and anticancer activities, as well as potent free radical scavenging compounds [5], attributed to highly bioactive polyphenols, ranging from simple molecules like flavonoids to more complex compounds like tannins, which highlights *Limonium* species as promising sources for the development of pharmaceuticals and nutraceuticals. On the Portuguese coast, there are about 17 *Limonium* species, including the Iberian endemic species *Limonium algarvense* Erben, which is scattered amongst the provinces of the Algarve (Portugal), Huelva and Cadiz (Spain), but is also found in south Morocco [1,6,7]. *L. algarvense* has been studied over the last 8 years as a promising and rich source of bioactive phenolic compounds, such as phenolic acids (salicylic, gallic, gentisic and coumaric acids) and flavonoids (catechins, quercetin, apigenin, luteolin, naringenin and their glycoside derivatives), which have been related to strong biological effects, mainly antioxidant and anti-inflammatory, but also anti-melanogenic, neuroprotective and antidiabetic properties [8,9,10,11,12].

Human’s natural metabolism produces harmful free radicals that, when not neutralized by natural antioxidant defense systems, can attack cell macromolecules impairing normal cellular processes such as enzyme activity, cell division and energy production, leading to chronic oxidative stress [13]. This state is associated with the development of several pathological conditions, including inflammation, metabolic disorders, cancer, diabetes, neurodegeneration, and cardiovascular problems [14]. Thus, exogenous antioxidants, such as phenolic compounds, can thus have a role in protecting organisms from the oxidative damage by scavenging free radicals, reducing molecules, and chelating metals, converting oxidants into more stable compounds [15]. For that, spectrophotometric methods were privileged for estimating the antioxidant potential of new products due to their affordability, simplicity, reproducibility, cost-effectiveness, and rapidness. Despite potential drawbacks like lack of specificity or differing reactivities, they remain valuable for making relative comparisons between samples in initial assessments and exploratory research [16]. Also, chronic inflammation-mediated diseases are one of the most significant threats to human health, being fatal to 3 out of 5 people worldwide, including cancer, diabetes, cardiovascular and pulmonary diseases, and obesity [17]. One of the main players of chronic inflammatory response is nitric oxide (NO) that is mainly generated by activated macrophages [18]. Thus, natural anti-inflammatory agents can play a role in shielding organisms from inflammatory damage by reducing macrophages NO production.

The synthesis of bioactive metabolites is triggered by several environmental conditions, including plant developmental stage, season, stresses, or nutrient availability [19], especially for halophytes such as sea lavenders that thrive in maritime salt marshes subjected to extreme abiotic variations [20]. As a consequence, the biological properties conferred by them can also vary according to these constraints [21]. For these reasons, various Pharmacopoeias recommend collecting seasons for medicinal plants to obtain the maximum therapeutic effect. Thus, the optimal season for harvesting medicinal plants from the wild should be determined to obtain the highest yields of bioactive molecules with medicinal interest [22]. Moreover, this knowledge could also be used to improve the yields of bioactive metabolites in domesticated plants by selecting and identifying top-producing ecotypes and defining the best environmental conditions (e.g., temperature, humidity, day length, UV exposure) for optimized crop productivity. To do this, plants collected in different geographic locations (different ecotypes) and in different seasons of the year (different climatic conditions and day lengths) can serve as a model to define the best conditions for the optimized production of bioactive molecules and biological properties [21,23]. However, despite *Limonium* being a medicinal genus, just a few studies have reported the effect of seasonality on the phytochemical profile and antioxidant properties, including for *L. pruinosum*, *L. tunetanum*, *L. delicatulum*, and *L. reniforme*, which showed elevated levels of phenolics, flavonoids, and tannins and prominent antioxidant activities were detected in plants harvested during the summer months [24]. In turn, as far as we know, the influence of geographical location (different ecotypes) on the chemical composition and bioactivities was never studied in any *Limonium* species.

**Figure 1:**
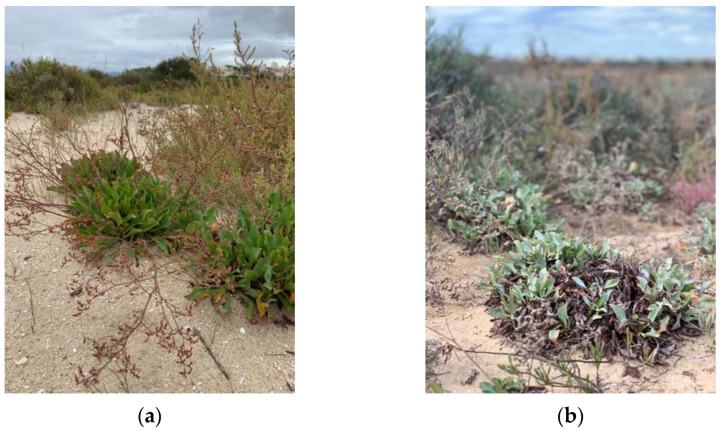
*Limonium algarvense* (sea lavender) in Southern Portugal: (**a**) Ria de Alvor Lagoon (spring), and (**b**) Ria Formosa lagoon (autumn). Photos by Catarina Guerreiro Pereira.

In this context, this work aimed to evaluate the influence of both site and season of collection of sea lavender plants on its metabolites and biological properties. For that purpose, biomass was collected from two different locations in southern Portugal, namely western (Barlavento: “Ria de Alvor”, Portimão [Figure 1a] and eastern (Sotavento: “Ria Formosa”, Tavira [Figure 1b] lagoons) areas during winter, spring, summer, and autumn. Aqueous acetone extracts were then prepared from dried biomass and evaluated for their total contents in phenolics, flavonoids and tannins, and for detailed phytochemical profile by liquid chromatography electrospray ionization quadrupole time-of-flight mass spectrometry (LC-ESI-QTOF-MS/MS). Samples were also appraised for in vitro antioxidant and anti-inflammatory properties.

## 2. Results and Discussion

### 2.1. Phenolic Composition

**Figure 2:**
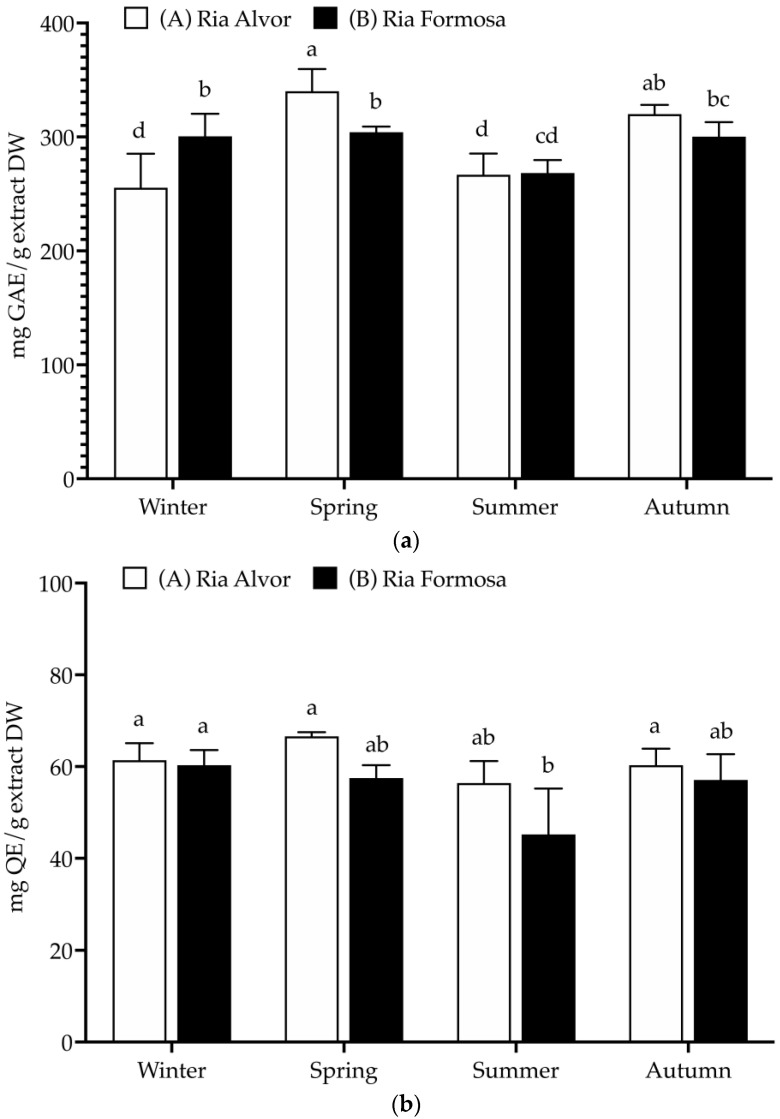

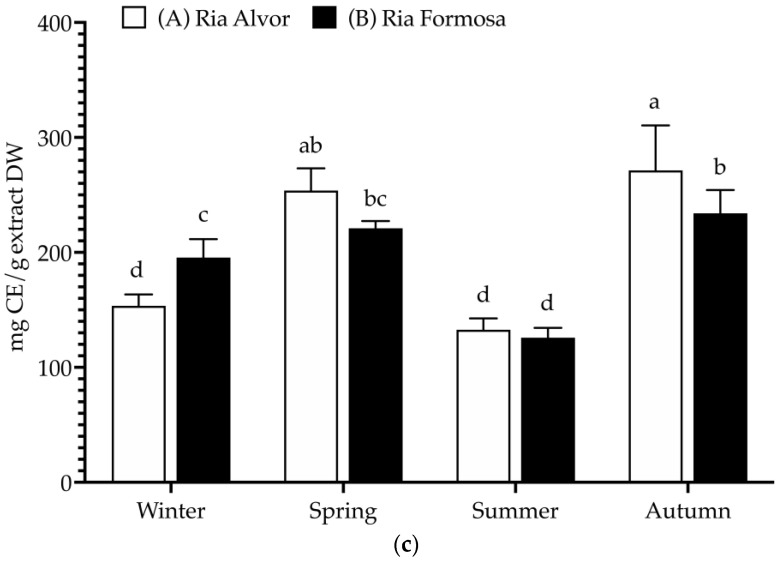
Total polyphenolic contents (mg/g extract, DW) of *Limonium algarvense* (sea lavender) extracts prepared from biomass collected in the two locations: (**a**) Total phenolic content (TPC), (**b**) Total flavonoid content (TFC), and (**c**) Condensed tannins content (CTC). Results represent the mean ± SD (*n* = 6). Different letters represent significant differences (*p* < 0.05).

In this work, plants collected from different geographic locations (Barlavento: Ria de Alvor” and Sotavento: Ria Formosa”) and seasons (spring, summer, autumn, and winter) were evaluated for their total phenolics, flavonoids and tannins contents (Figure 2).

A high TPC was found in all samples (>255 mg GAE/g DW), being significantly increased during spring in “Ria de Alvor” (340.2 ± 19.4 mg GAE/g DW), in contrast to plants collected in summer from both locations (A: 266.9 ± 18.5 and B: 268.4 ± 11.4 mg GAE/g DW). Generally, the accumulation of phenolics was higher in spring and autumn in “Ria de Alvor” plants, whereas during winter it was lower than in “Ria Formosa” ones. In turn, summer samples did not exhibit significant differences amongst locations (*p* < 0.05). Regarding flavonoid content, there were no significant differences between seasons and geographical variations (approx. 60 mg QE/g DW), except for plants collected in “Ria Formosa” during the summer that presented reduced levels of flavonoids (45.2 ± 10.0 mg QE/g DW). Contrarily, the tannin levels showed high seasonal variability as well as geographical variations. For instance, plants collected during autumn in “Ria de Alvor” had the highest tannin content (271.3 ± 39.2 mg CE/g DW), followed by samples collected in spring in the same location (253.8 ± 19.4 mg CE/g DW). When collected in summer, samples present lower levels of tannins, regardless of geographical location (A: 132.7 ± 9.9 and B: 125.8 ± 8.5 mg CE/g DW). In addition, the samples from “Ria de Alvor” had generally higher tannin concentrations than those from “Ria Formosa”, with the exception of the winter samples, which showed an opposite trend. 

The levels of phenolics and tannins in sea lavender are influenced by seasonal and geographical variations, consistent with the fact that the accumulation of phenolics by plants may be affected by different environmental conditions, such as climate and location [25]. Therefore, the utmost levels perceived in spring and autumn seasons may be a result of the plant’s response to the environmental conditions during these seasons, such as optimal mild temperatures in spring and autumn (24.7–20.0 °C), while the extremely high temperatures during summer can cause increased heat stress that may cause impaired plant growth, photosynthesis, reproduction, yield and reduced primary and secondary metabolism [26,27]. Likewise, the cold stress occurring during the winter season is also a main abiotic stress factor with strong effects on plant growth and development, significantly impacting the synthesis of plant metabolites [28]. These reasons may justify the lowest contents of phenolics and tannins in plants collected during summer and winter when compared to spring and autumn.

Moreover, in spring the aerial parts included the mature inflorescences, which also could influence the variations observed in the present study; as previously described by Rodrigues et al. [8], the flowers of sea lavender contain the highest levels of phenolics compared to the other organs. Additionally, in autumn sea lavender plants produce mature seeds, which may also be associated with highest content in tannins typically found in seeds coat to reduce their permeability and contribute to keep seed dormancy [29]. While a direct comparison cannot be made since there are no previous studies on the total polyphenols of whole aerial parts, when comparing the results obtained in this work with previously published data on this species, it is possible to observe that flowers had similar levels of total phenolics (228 mg GAE/g DW), while total flavonoids were superior (236 mg QE/g DW) and tannins levels were lower (145 mg CE/g DW). The remaining aerial organs (leaves and peduncles) presented lower contents of phenolics (54–83 mg GAE/g DW), flavonoids (44–51 mg QE/g DW) and tannins (14–19 mg CE/g DW) [8]. 

Moreover, there are a few studies on the effect of seasonal effects on the phytochemical composition of other species of the same genus. For instance, *L. reniforme* from Iran was evaluated for phytochemical changes, enzymatic activities, and antioxidant activities along the different seasons, where phenolics and flavonoids exhibited a significant increase in the summer compared to other seasons, which were attributed to the activation of physiological and biochemical processes related to salinity adaptation, such as increased antioxidant enzymatic activities (peroxidase, catalase, polyphenol oxidase, ascorbate peroxidase) [24]. In turn, in a study focusing on *L. delicatulum* within Tunisian Sabkha, researchers explored potential correlations between phenolics and flavonoids accumulation with stress response mechanisms (ion uptake, activation of antioxidant systems), soil parameters and climatic data throughout the year. They have found that the accumulation of phenolics and flavonoids was observed during the dry period (summer), which was correlated to the activation of plant antioxidant systems to counteract extreme conditions like high salinity, drought, nutritional deficiency, and high temperatures [30]. Mahmoudi et al. [31] investigated the effect of seasonality on the phytochemical profile and antioxidant potential of Tunisian *L. pruinosum* and *L. tunetanum*, aiming to establish the optimum harvesting time. Concomitantly, the shoots harvested during the dry season had a significantly higher content of bioactive compounds and, consequently, greater antioxidant activity compared to those collected during the wet period. Once again, a dry season seems to induce a stress response in these plants, as evidenced by high levels of oxidative stress markers like lipid peroxidation, hydrogen peroxide, and electrolyte leakage. In fact, is well known that phenolic compounds are synthesized in response to abiotic stresses like salinity, drought, and excessive solar radiation, playing a key role in protecting plant cells from reactive oxygen species (ROS) by neutralizing their harmful effects and adjusting lipid peroxidation kinetics. This helps maintain membrane integrity and supports photosynthetic processes by reducing damage to photosynthetic systems by absorbing UV radiation [32].

However, these findings were the opposite to those obtained in the present study, where the highest levels of phenolics were obtained in spring and autumn, indicating that the aforementioned differences may be related to different years of collection, or to interspecific variations, but appear to be more strongly associated with organ-related factors, i.e., whole aerial parts versus separate organs, since the presence of flowers and seeds appears to be definitely linked to the higher concentrations of secondary metabolites found in these samples, as already reported in previous studies on this species [8,12].

**Figure 3:**
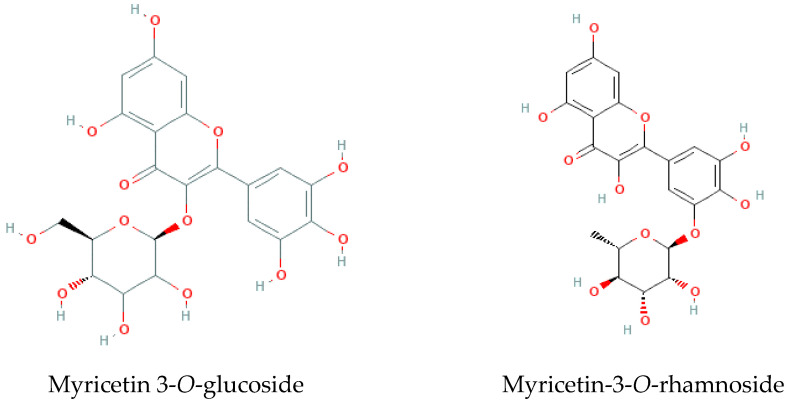
Two of the major molecules identified in *Limonium algarvense* (sea lavender) extracts (Table 1; adapted from PubChem).

Aiming to putatively identify and quantify the individual phenolic components present in the extracts, samples were further analyzed by LC-MS/MS, and results are presented in Table 1 and Figure 3. Around 18% of the tentatively identified compounds were represented by myricetin glycosides (peaks No. 21–25, 28, 29, 34, 46, 50, 51, i.e., 10 of 55 detectable peaks). Other compounds included lignans (peaks 35, 36, 39, 43–45, 48, 49), such as the most abundant pinoresinol sulphate and a few minor derivatives, polyol (inositol) derivative, galactinol, as well as a few other flavonoids, none of which in amounts comparable to the major myricetin glycosides. 

It is interesting to note that some compounds were only detected in particular seasons and locations or had increased or decreased amounts among these variables. For example, luteolin (52), naringenin (53), and two apigenin derivatives (41, 42) were found in increased concentrations in the summer samples, whereas a myricetin derivative (50) was detected in higher amounts during the spring. In turn, prodelphinidinA2 3′-gallate (15) was detected in higher amounts during autumn and winter, while syringaresinol sulphate (35) and medioresinol sulphate (36) have lower concentrations during the spring, and quercetin-*O*-hexoside isomer (37) is only produce in summer. Concerning the different locations, myricetin-3-*O*-rhamnoside (29) and theasinensin B (13) have shown higher abundance in samples collected in “Ria Formosa”, while galactinol dihydrate (3) was present in a larger amount in plants from “Ria de Alvor”.

High-performance liquid chromatography with diode-array detection (HPLC-DAD) and liquid chromatography tandem high-resolution mass spectrometry (LC-HRMS/MS) had already identified most of these molecules in the aerial parts (flowers, peduncles, or leaves) of this species, either collected from the wild or cultivated in a greenhouse [8,10] (Rodrigues et al., 2019, 2021). Except galactinol (2), galactinol dihydrate (3), myricetin-3-*O*-(3-caffeic acid-glucoside) (33), and two lignan glycosides (48, 49) that are here reported for the first time in sea lavender extracts. 

Flavonoids, such as luteolin, naringenin, apigenin, and myricetin and their derivatives, have a multitude of functions in plants, including regulating plant development, pigmentation, UV protection, defense and signaling between plants and microorganisms [33]. Therefore, their highest occurrence in summer and spring samples may be related to these roles; for example, apigenin and luteolin glycosides have been strongly correlated with increasing UV-B levels [34], while other flavonoids can act as major pigments in the flowers of higher plants, contributing to attract pollinators and seed dispersion [35]. Moreover, these molecules often provide medicinal properties to the plants, such as antioxidant, anti-inflammatory and anti-microbial properties, having multiple applications as pharmaceutical and cosmetic ingredients, as well as in the food industry as preservatives, pigments, and antioxidants [35]. Moreover, other major detected compounds, like myricetin glycosides and pinoresinol, are known as antioxidant and anti-inflammatory agents [36,37], while galactinol, a plant intracellular antioxidant stress defense signal, has been discovered as a skincare ingredient [38], and lignans, like syringaresinol and medioresinol, related to defensive mechanisms against external agents, are also reported with physiological properties such as antioxidant, phytoestrogen, or anticancer [39]. These results shed new light on the chemical richness of sea lavender and highlights its potential as a source of molecules suitable for the development of new natural products with potential health benefits. 

Additionally, as “Ria Formosa” and “Ria de Alvor” generally share similar climatic characteristics, as they are both coastal wetland areas in southern Portugal, the differences found among the locations may be linked to genetic variation factors within different populations/ecotypes. These genetic variations could contribute to the distinct biochemical compositions observed, a phenomenon documented in other plant species, such as in the fruits of different ecotypes of the Tunisian halophyte *Eryngium maritimum* [40], as well as common glycophytes like *Cichorium spinosum* [41], or *Trigonella monantha* [42]. This suggests that genetic diversity may play a role in shaping the chemical profiles of *L. algarvense* in varied ecological niches. Overall, the variations among the produced compounds may be linked to different genetics and metabolism patterns, reflecting the plant’s needs throughout different seasons and ecological challenges.

**Table 1:** Liquid chromatography electrospray ionization quadrupole time-of-flight mass spectrometry (LC-ESI-QTOF-MS/MS) analysis with tentative annotation of major phenolic compounds of *Limonium algarvense* (sea lavender) collected throughout the year (four seasons) in the two locations (A—“Ria de Alvor”; and B—“Ria Formosa”; Figure 2).

ID	Rt (min)	Proposed Ion Structure (M-H)^−^	[M-H]^−^ [m/z (Δ ppm)]	MS^2^ Main-Ion [Relative Intensity (%)]	Proposed Compound	Winter		Spring		Summer		Autumn		Ref.
						A	B	A	B	A	B	A	B	
1	0.9	-	272.9472	180.9572 (100)	Unknown	+	+	+	+	+	+	+	+	[12]
2	0.9	C_12_H_21_O_11_	341.1087 (+4.5)	-	Galactinol	+	+	+	+	+	+	+	+	[43]
3	1.0	C_12_H_21_O_11_**·** _2_(H_2_O)	377.0875, 341.1074 (+4.5) [M-H-2(H_2_O)]^−^	341.1074 (100), 179.0569 (20)	Galactinol dihydrate	++	+++	+++	+++	++	++	+++	+++	[44]
4	1.5	C_13_H_9_O_8_	293.0311 (−2.7)	121.0502 (100)	Pyrogallol gallate	+	+	+/−	+/−	+/−	+/−	+/−	+/−	[12]
5	2.6	C_13_H_15_O_10_SO_3_	411.0221 (+4.3)	240.9997 (100), 331.0607 (34) 169.0134 (13)	Glucogallin sulphate	+	+	+/−	+/−	+/−	+/−	+	+	[12]
6	3.4	C_15_H_19_O_10_SO_3_	439.0535 (+3.8)	241.0115 (100) 198.0794 (41)	Glucosyringic acid sulphate	+/−	+/−	+/−−	+/−−	+/−	+/−	+/−	+/−	[12]
7	4.7	C_15_H_17_O_8_SO_3_	405.0480 (+4.2)	240.9830 (80) 341.9238 (31)	Glucosyl coumaric acid sulphate	+	+	+	+	+	+	+	+	[12]
8	5.1		365.0151	210.9808 (100) 97.0595 (48) 139.1229 (38)	Unknown	+	+	+/−	+/−	+/−	+/−	+	+	
9	6.5	C_15_H_17_O_8_SO_3_	405.0488 (+2.2)	240.9992 (100) 97.0492 (61) 271.044 (42)	Glucosyl coumaric acid sulphate isomer	+	+	+	+	+	+	+	+	[12]
10	7.6		259.0275	166.6115 (100)	Unknown	+/−−	+/−	+/−−	+/−−	−	+/−−	+/−−	+/−−	
11	8.4		441.1602	174.9519 (100), 381.1242 (72), 276.9178 (67)	Unknown	−	+/−	−	+/−	−	+/−	−	+/−	
12	8.5		463.1427	293.0865 (100), 348.8434 (63), 315.9599 (56)	Unknown	+/−	−	+/−	−	+/−	−	+/−	−	
13	9.2	C_37_H_29_O_18_	761.1366 (−0.9)	423.0676 (100), 305.0622 (71), 609.1168 (24)	Theasinensin B	+/−	+	+/−	+	+/−	+	+/−	+	[12]
14	9.8	C_17_H_29_O_10_	393.1741 (+6.4)	179.0441 (100), 205.0639 (71), 197.4096 (67)	Hex-3-en-olxylopyranosyl- (1-6)-glucopyranoside	−	−	−	−	++	+	−	−	[10]
15	10.3	C_37_H_27_O_18_	759.1191 (+1.6)	423.0714 (100), 301.0297 (67), 345.0175 (63), 481.0677 (33)	ProdelphinidinA2 3′-gallate	+	+	+/−	+/−	+/−	+/−	+	+	[12]
16	11.3	C_21_H_21_O_11_	449.1062 (+6.1)	287.0521 (100), 269.0352 (92)	Eriodyctiol-*O*-glucoside	+/−−	+/−−	+/−−	+/−−	+/−−	+/−−	+/−−	+/−−	[10]
18	12	C_22_H_17_O_11_	457.0758 (+4.0)	305.0611 (100), 169.0127 (64)	Epigallocatechin gallate	+	+	+	+	+	+	+	+	[10]
19	12.5		385.1116	267.0685 (100)	Unknown	+/−	+/−	+/−	+/−	+/−	+/−	+/−	+/−	
20	12.6		431.1909		Unknown	+/−	−	+/−	−	+/−	−	+/−	−	
21	12.9	C_28_H_23_O_17_	631.0941 (−0.1)	479.0799 (100), 316.0175 (55)	Myricetin-3-*O*-galloyl-hexoside	+++	+++	+++	+++	+++	+++	+++	+++	[10,12]
22	13.5		539.2144	491.1935 (100), 195.0645 (32), 329.1318 (22), 343.1460 (20)	Unknown	+/−	+/−	+/−	+/−	+/−	+/−	+/−	+/−	
23	13.6	C_27_H_29_O_17_	625.1397 (+2.1)	316.0154 (100), 287.0172 (20), 271.01630 (17)	Myricetin-3-*O*-rutinoside	+/−	+	+	+/−	+/−	+/−	+/−	+	[12]
24	13.7	C_21_H_19_O_13_	479.0820 (+2.3)	316.0187 (100), 271.0211 (6)	Myricetin-*O*-glucoside	+++	+++	+++	+++	+++	+++	+++	+++	[10,12]
25	13.9	C_21_H_19_O_13_	479.0823 (+1.7)	316.0181 (100), 271.0204 (5)	Myricetin-*O*-glucoside (isomer)	++	++	++	++	++	++	++	++	[10,12]
26	14.8	C_28_H_23_O_16_	615.0991 (0)	463.0846 (100), 300.0238 (30), 301.0309 (21), 271.0202 (15)	Quercetin-3-*O*-galloyl-hexoside	+/−	+/−	+/−	+/−	+/−	+/−	+/−	+/−	[10,12]
27	15.1	C_28_H_23_O_16_	615.0993 (−0.2)	463.0870 (100), 300.0227 (37), 301.0325 (36), 271.0206 (36)	Quercetin-3-*O*-galloyl-hexoside (isomer)	+	+	+	+	+	+	+	+	[10,12]
28	15.3	C_20_H_17_O_12_	449.0707 (+4.2)	316.0186 (100), 317.0227 (34), 271.0192 (9)	Myricetin-3-*O*-pentoside	+/−	+/−	+/−	+/−	+/−	+/−	+/−	+/−	[10]
29	15.5	C_21_H_19_O_12_	463.0866 (+3.5)	316.0179 (100), 317.0232 (33), 271.0180 (7)	Myricetin-3-*O*-rhamnoside	++	+++	++	+++	++	+++	++	+++	[10,12]
30	15.7	C_21_H_19_O_12_	463.0870 (+2.7)	300.0236 (100), 301.0285 (41), 316.0180 (26), 271.0208 (11)	Quercetin-*O*-hexoside	+	+	+	+	+	+	+	+	[10]
31	15.9	C_27_H_33_O_15_	597.1818 (+1.1)	387.1077 (100), 357.0951 (92), 417.1151 (35), 459.1230 (11)	Quercetin-tetramethyl ether-dihydroxyethyl-fructopyranose	+/−	+/−	+/−	+/−	+/−	+/−	+/−	+/−	[10]
32	16.1	C_21_H_19_O_12_	463.0866 (+3.5)	300.0216 (100), 301.0305 (52), 316.0177 (28), 271.0192 (7)	Quercetin-*O*-hexoside isomer	+	+	+	+	+	+	+	+	[10]
33	16.2	C_30_H_27_O_17_	659.1253 (+0.1)	316.0178 (100), 317.0225 (29), 287.0164 (13), 271.0175 (13)	Myricetin-3-*O*-(3-caffeic acid-glucoside)	+/−−	+/−−	+/−	+/−	+/−	+/−	+/−−	+/−−	
34	16.4	C_18_H_17_O_7_SO_3_	425.0530 (+4.2)	300.0608 (100), 315.0797 (48), 345.1509 (26)	3′,4′,5′-Trimethoxyflavanone sulphate	+	+	+	+	+	+	+	+	[12]
35	16.6	C_22_H_25_O_8_SO_3_	497.1116 (+1.4)	417.1541 (100), 402.1316 (44), 418.1583 (24), 387.1017 (18) 181.0492 (16)	Syringaresinol sulphate	++	++	+	+	++	++	++	++	[12]
36	16.6	C_21_H_23_O_7_SO_3_	467.1001 (+3.5)	387.1383 (100), 372.1181 (73), 181.0508 (30), 357.0943 (23)	Medioresinol sulphate	+	+	+/−	+/−	+	+	+	+	[12]
37	16.8	C_21_H_19_O_12_	463.0868 (+3.1)	301.0292 (100), 300.0206 (44)	Quercetin-*O*-hexoside isomer	−	−	−	−	+	+/−	−	−	[12]
38	17.3		583.1482	309.0322 (91), 322.8592 (86), 291.0268 (77), 65.7793 (77)	Unknown	+/−	+/−	+/−	+/−	+/−	+/−	+/−	+/−	
39	17.7	C_20_H_21_O_6_SO_3_	437.0905 (+1.6)	357.1322 (100), 342.1076 (53), 422.0608 (10), 151.0454 (3)	Pinoresinol sulphate	+++	+++	+++	+++	+++	+++	+++	+++	[10,12]
40	17.9	C_21_H_19_O_11_	447.0921 (+2.6)	300.0229 (100), 301.0306 (66), 271.0188 (12)	Quercetin-3-*O*-rhamnoside	+	+	+	+	+	+	+	+	[10]
41	18.1	C_21_H_19_O_10_	431.0966 (+4.1)	268.0334 (100), 269.0405 (40)	Apigenin-*O*-glucoside	−	−	−	−	++	+	+/−	+/−	[10]
42	18.4	C_21_H_17_O_11_	445.0755 (+4.8)	269.0404 (100)	Apigenin-*O*-glucuronide	−	−	−	−	+++	++	+	+	[10,12]
43	18.6	C_27_H_35_O_12_	551.2130 (+0.6)	357.1265 (100)	Pinoresinol derivative	+/−	+/−	+/−	+/−	−	−	+/−−	+/−−	[12]
44	18.9	C_20_H_21_O_6_SO_3_	437.0897 (+3.3)	357.1283 (100), 342.1063 (92)	Pinoresinol sulphate isomer	+	+	+	+	+	+	+	+	[10,12]
45	19.7	C_27_H_35_O_12_	551.2137 (−0.5)	357.1226 (100), 165.0621 (95), 195.0632 (92), 438.8498 (38), 505.2076 (34)	Pinoresinol derivative	+	+	+	+	+	+	+	+	[12]
46	19.9	C_28_H_23_O_16_	615.0992 (−0.1)	317.0261 (100), 287.0180 (3), 463.0856 (3)	Myricetin-*O*-(galloyl)-deoxyhexose	+/−	+	+	+	+	+	+	+	[10]
47	20	C_21_H_19_O_10_	431.0973 (+2.5)	284.0341 (100), 255.0201 (32)	Luteolin-7-*O*-rhamnoside	+/−	+/−	+/−	+/−	+	+/−	+/−	+/−	[10]
48	20.5	C_27_H_33_O_12_	549.1978 (−0.1)	355.1193 (100), 521.2029 (84), 193.0412 (73)	Lignan glycoside	+	+	+	+	+/−	+/−	+	+/−	[45,46]
49	21	C_26_H_31_O_10_	503.1924 (−0.3)	335.4861 (100), 306.8672 (80), 426.7623 (65)	Lignan glycoside	+/−	+/−	+/−	+/−	+/−−	+/−−	+/−	+/−	[47]
50	22.3	C_25_H_23_O_13_	531.1122 (+4.2)	316.0170 (100), 317.0207 (29), 271.0193 (6), 287.0174 (5)	Myricetin derivative	+/−−	+/−	+	+	+/−	+/−	−	−	
51	22.5	C_26_H_27_O_14_	563.1421 (−2.6)	316.0197 (100), 317.0233 (24)	Myricetin derivative	+/−−	+/−	+/−−	+/−	+/−−	+/−	+/−−	+/−−	
52	22.9	C_15_H_9_O_6_	285.0381 (+8.2)	124.1813 (91), 180.9969 (91), 216.9228 (74)	Luteolin	−	−	−	−	+/−	−	−	−	[12]
53	25.4	C_15_H_11_O_5_	271.0590 (+8.0)	85.0673 (100), 150.5868 (35)	Naringenin	−	−	−	−	+	+	+/−−	+/−	[10,12]
54	26.2	C_18_H_31_O_5_	327.2161 (+5.0)	283.2492 (100)	Trihydroxy-10,15-octadecadienoic acid	+/−	+/−	+/−	+/−	+	+/−	+/−	+/−	[10,12]
55	28.2	C_18_H_33_O_5_	329.2313 (+6.3)	-	Trihydroxy-10-octadecenoic acid	+/−	+/−	+/−	+/−	+/−	+/−	+/−	+/−	[10,12]

Retention times, MS data, and ion formula suggestion of the constituents present in the extracts of *L. algarvense*. For distinguishing amongst trace, very low, low, medium, or high abundance the symbols +/−−, +/−, +, ++ and +++ were used, respectively.

### 2.2. Antioxidant Properties

Similarly to the phenolic composition, the antioxidant capacity of the plants can be affected by different environmental conditions, and, in this context, sea lavender extracts were evaluated for their radical-scavenging activity towards DPPH, ABTS and NO, ferric reducing capacity and copper and iron chelating activities (Table 2).

Similarly to the phenolic composition, the spring and autumn samples from “Ria de Alvor” (A) were the most active towards DPPH• (EC_50_ = 44.5–45.6 µg/mL) and in chelating copper (EC_50_ = 176–184 µg/mL), closely followed by plants from “Ria Formosa” (B) collected in the same seasons (DPPH: EC_50_ = 62.7–65.6 µg/mL; CCA: EC_50_ = 185–193 µg/mL). In contrast, the samples collected during winter were the most active on ABTS•+ (A: EC_50_ = 26.9 µg/mL; B: EC_50_ = 19.6 µg/mL), as well as towards NO• (B: EC_50_ = 68.0 µg/mL). Regarding the ferric reducing capacity, autumn samples presented the lowest EC_50_ values, similar in both locations (EC_50_ = 61.3–63.3 µg/mL). None of the samples were able to chelate iron up to the concentration of 1000 µg/mL. These dissimilarities highlight the importance of using different in vitro methods for a more reliable assessment of the antioxidant activity of a sample.

Rodrigues et al. [8] reported an EC_50_ value of 90 µg/mL towards DPPH• for a methanol extract of flowers of the same species collected in June in Ria de Alvor, which is slightly higher than those obtained in this study for the spring samples (45.6–62.7 µg/mL). Likewise, the obtained EC_50_ values for ABTS•+ (270 µg/mL) were also higher in comparison to our results for all samples (26.9–118 µg/mL). In relation to copper chelating potential, the activity of methanol extracts from flowers (EC_50_ = 290 µg/mL mg/mL) was a little higher than that obtained in this work for the spring season (EC_50_ = 176 µg/mL). Nevertheless, the same flower and leaf samples presented much lower EC_50_ values on FRAP (10 and 18 µg/mL, respectively) when compared to the plants collected in summer or spring in the present study (EC_50_ = 240–333 µg/mL). Although methanol extracts from different organs of the sea lavender have already been tested previously [8], this is the first time that extracts of this species have demonstrated the ability to scavenge the NO radical. In addition, like for the total polyphenol content, and contrasting with what was observed in this study, the DPPH• scavenging activity of *L. reniforme*, *L. delicatulum*, *L. pruinosum,* and *L. tunetanum* samples was higher during the dry (summer) season, related to stress-induced activation of physiological and biochemical processes, such as increased antioxidant enzymatic defense systems [24,25,26]. This discrepancy underscores the complexity of seasonal influences on antioxidant activities within the *Limonium* genus, and as discussed above, the different patterns are possibly associated with variations in environmental conditions, species genetic variability, and the inclusion of different plant organs. Having in mind that a correlation between phenolic compounds and antioxidant activity has already been broadly explored in the literature, the generally superior antioxidant activity noted in spring and autumn samples is probably correlated to the increased phenolic and tannin content found during these seasons. 

### 2.3. Anti-Inflammatory Properties

**Figure 4:**
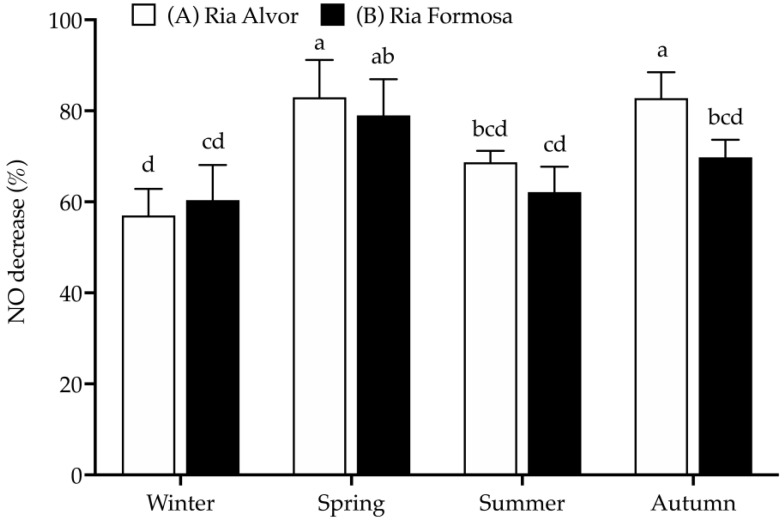
Anti-inflammatory activity (% NO decrease) of *Limonium algarvense* (sea lavender) extracts (25 µg/mL) prepared from biomass collected in the different locations and seasons. L-Name (200 µg/mL) was used as positive control (NO decrease: 73.4 ± 4.3% ^abc^). Results represent the mean ± deviation (SD) (*n* = 9). Different letters (a–d) represent significant differences (*p* < 0.05).

In this work, RAW 264.7 macrophages were stimulated with LPS to increase NO production, simulating an inflammatory response, and the ability of sea lavender extracts to reduce NO production was evaluated at a concentration of 25 µg/mL, as it was the only one that did not present significant toxicity in RAW 264.7 macrophages (>80% cell viability (Figure 4).

As already seen for the antioxidant activity (DPPH• and CCA), the spring and autumn samples from “Ria de Alvor” (A) were the most effective in reducing NO production (approx. 83%), along with the plants from “Ria Formosa” (B) collected during spring (approx. 80%). The winter samples showed an opposite trend, presenting the lowest NO decrease (approx. 58–60%). Additionally, the samples collected in “Ria de Alvor” are generally more active than those from “Ria Formosa”, except for winter. 

Infusions and decoctions of composite samples containing sea lavender flowers collected in June from different sites (Ludo, Vilamoura and Castro Marim) have already demonstrated the ability to reduce NO production in LPS-stimulated macrophages, with EC_50_ values ranging between 46.3 and 48.5 µg/mL [9], but in the present work we observed a higher NO decrease at a lower concentration. This difference can be related to different plant organs, extraction solvents and methodologies, seasonal and/or year of collection [48]. Once again, it is possible to perceive that the anti-inflammatory activity fluctuates according to the content of total phenols and tannins. For instance, samples richer in phenolics and tannins (spring and autumn) had higher activity, while less active samples (winter and summer) presented lower amounts of phenols and tannins, indicating a possible correlation. Unfortunately, there were no previous studies in *Limonium* species on the seasonal or geographical dynamics of anti-inflammatory activities, while contrary to this study, no seasonal influence was observed for the anti-inflammatory properties of other halophyte species, *Cladium mariscus* from “Ria Formosa” [49].

### 2.4. Principal Component Analysis (PCA)

**Figure 5:**
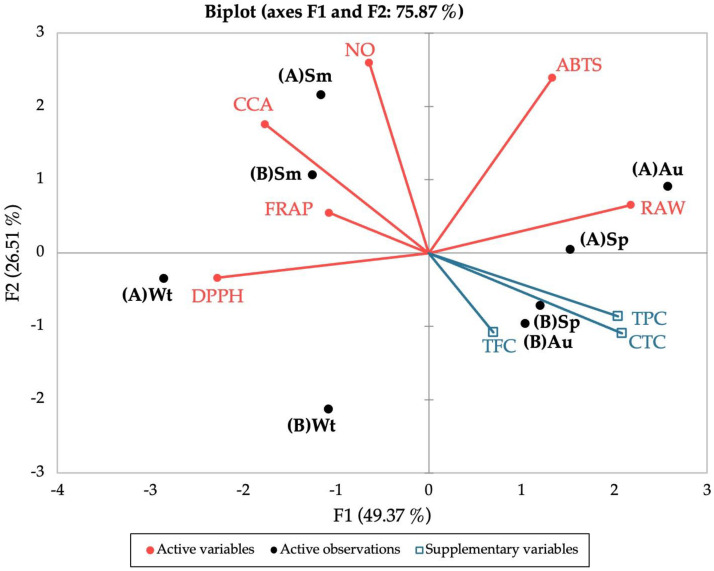
Principal Component Analysis (PCA) of the antioxidant (DPPH, ABTS, NO, FRAP, CCA), anti-inflammatory (RAW) and polyphenolic contents (TPC, TFC, CTC) of the extracts prepared from biomass collected from different locations and seasons. (A) “Ria de Alvor”, (B) “Ria Formosa”; Wt: winter, Sp: spring, Sm: summer, Au: autumn.

To evaluate the effect of different seasons (winter, spring, summer, and autumn) and the influence of the site of collection (“Ria de Alvor” and “Ria Formosa”) in the biological properties and chemical composition of sea lavender, a biplot PCA was constructed with the sample scores and the variable loadings based on all parameters studied, namely antioxidant (DPPH•, ABTS•+, NO•, FRAP, CCA), anti-inflammatory activity, polyphenolic contents (TPC, TFC, CTC), geographical location (“Ria de Alvor” and “Ria Formosa”), and seasons (winter, spring, summer, and autumn) (Figure 5). 

The PCA described 75.87% of the total variation in the dataset. According to the results obtained in the biplot, there is a clear separation between samples from summer and winter from both locations, whereas plants collected during spring and autumn showed higher similarities according to the site of collection. For winter, samples from “Ria de Alvor” (A) showed high proximity to the DPPH• scavenging activity, while those from “Ria Formosa” (B) are distanced from all variables, pointing to a negative relation between this site/season and the studied variables. For summer, samples from both locations are in great proximity to FRAP, CCA, and NO• antioxidant activities, implying a stronger influence of these antioxidant parameters in this season. In turn, spring and autumn samples collected in “Ria de Alvor” (A) are closely related to improved anti-inflammatory activity, while samples from “Ria Formosa” (B) seem to have greater influence from polyphenolic groups. Overall, plants from “Ria de Alvor” seem to gather the best conditions for the optimized production of bioactive molecules, coupled with environmental conditions during spring and autumn that appear to induce the highest biological properties of those samples. 

## 3. Materials and Methods

### 3.1. Sample Collection and Processing

**Figure 6:**
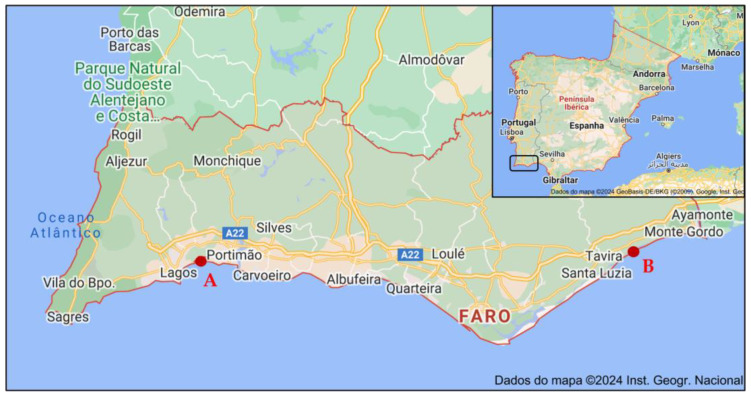
Locations of *Limonium algarvense* (sea lavender) harvesting in southern Portugal: (A) Barlavento: Ria de Alvor Lagoon, Portimão, (B) Sotavento: Ria Formosa Lagoon, Tavira. Adapted from Instituto Geográfico Português^®^2024.

The sampling approach involved collecting 10 specimens distributed along a 4 m transect of the saltmarsh, covering the range from the lowest marsh area, where initial specimens were located, to the highest marsh zone where they were no longer present. The whole aerial parts (comprising leaves, peduncles, flowers and seeds, when present; voucher code XBH01, XtremeBio lab. Herbarium, Faro, Portugal) from the 10 specimens were pooled together to form a composite sample. The collection timepoints were winter (January, 0 days), spring (May, 98 days), summer (July, 84 days), and autumn (November, 96 days) of 2020 (Table 3), in 2 distinct locations in southern Portugal (Figure 2): (A) Western area: Barlavento, Ria de Alvor Lagoon, Portimão (37°07′33.9″ N 8°35′52.8″ W), and (B) Eastern area: Sotavento, Ria Formosa Lagoon, Tavira (37°07′52.1″ N 7°36′38.5″ W). The locations selected (Figure 6) are in two lagoons with different characteristics and of considerably different sizes (site A, Ria de Alvor: ~15 km^2^, small shallow estuary [50]; site B, Ria Formosa: ~100 km^2^, multi-inlet barrier system [51], roughly 70 km apart. Samples were oven-dried at 40 °C in forced convection for 3 days, powdered, and stored at −20 °C until needed. 

### 3.2. Extracts Preparation

Dry biomass samples were extracted with 80% aqueous acetone (1:40, *w*/*v*) after stirring for 24 h, at room temperature (RT, approx. 20 °C). Extracts were filtered (paper Whatman nº4), solvent evaporated under reduced pressure (at 40 °C in rotary evaporator R-210, Buchi Labortechnik AG, Flawil, Switzerland), redissolved at 25 mg/mL in dimethyl sulfoxide (DMSO), and stored at −20 °C until needed.

### 3.3. Phytochemical Profiling

#### 3.3.1. Contents in Total Phenolic (TPC), Flavonoid, and Condensed Tannins (CTC)

The extracts’ contents in different phenolic groups were assessed by colorimetric assays, as fully described in Rodrigues et al. [8] and Oliveira et al. [49]. Briefly, the total phenolic content (TPC) was determined using the Folin–Ciocalteu reagent and absorbance measured at 725 nm (Biochrom EZ Read 400, Santa Clara, CA, USA) [8,49]; gallic acid was used as standard for the calibration curve (y = 1.294x + 0.038; R^2^ = 0.999), with results expressed as gallic acid equivalents (GAE). The total flavonoid content (TFC) was evaluated by the aluminum chloride (AlCl_3_) assay and absorbance measured at 415 nm [8,49]. Quercetin was the standard used (y = 2.148x + 0.056; R^2^ = 1.00), and results were expressed as quercetin equivalents (QE). The condensed tannin content (CTC) was assessed by the 4-dimethylaminocinnamaldehyde (DMACA) method, measuring absorbance at 640 nm [8,49]. The standard used was catechin (y = 0.361x + 0.037; R^2^ = 0.997), and results were expressed as catechin equivalents (CE). Results are presented as milligrams of standard equivalents per gram of extract dry weight (DW).

#### 3.3.2. Analysis by Liquid Chromatography Electrospray Ionization Quadrupole Time-of-Flight Mass Spectrometry (LC-ESI-QTOF-MS/MS)

Extracts at a concentration of 25 mg/mL in dimethyl sulfoxide (DMSO) were taken from the refrigerator and brought to RT. Then, 100 µL of each extract was taken and added to 900 µL methanol and 300 µL water. All extracts were filtered (0.22 µm) prior to analysis. Analyzes were executed in triplicate for three independent samples. Liquid chromatography—electrospray ionization-quadrupole time-of-flight (LC-ESI-QTOF) MS analysis was performed on a chromatographic system Thermo Dionex Ultimate 3000 RS (Thermo Fischer Scientific, Waltham, MA, USA), coupled to a QTOF mass spectrometer Bruker Compact (Bruker, Billerica, MA, USA). Separations were carried out on a column Kinetex C18 2.6 µm (150 mm × 2.1 mm), (Phenomenex, Torrance, CA, USA), maintained at 30 °C. Mobile phase A (H_2_O:HCOOH, 100:0.1, *v*/*v*) and B (acetonitrile:HCOOH, 100:0.1, *v*/*v*) were used in the following gradient program: 0–30 min 5–40% B, 30–32 min 40–95% B, 32–34 min 95% B followed by column equilibration with 5% B for 2 min between injections. The flow rate was 0.3 mL/min. AnalysisSpectra were acquired over a mass range from *m*/*z* 50 to 2000 (5-Hz) in negative-ion mode. ESI-MS conditions were as follows: splitless, nebulizer pressure 30 psi; dry gas flow 8 L/min; dry temperature 250 °C; and capillary voltage 4.5 kV. Mass spectra were recorded using scan range (*m*/*z*) 50–2200. The collision energy was set automatically from 10 to 95 eV, depending on the *m*/*z* of the fragmented ion. Processing of spectra was performed using the Bruker Data Analysis 4.3 software. 

### 3.4. Biological Properties

The biological activities of the samples were assessed at a concentration of 1 mg/mL, at five different concentrations (serial dilution from 10 to 0.01 mg/mL) and results were calculated as percentage (%) of activity relative to a negative control (samples’ solvent), except for the FRAP (ferric reducing antioxidant power) assay that was relative to the positive control. Whenever possible, the half maximal effective concentrations (EC_50_ values, mg/mL) were calculated. 

#### 3.4.1. In Vitro Antioxidant Activity

The radical scavenging activity (RSA) was assessed against the radicals DPPH• (1,1-diphenyl-2picrylhydrazyl), ABTS•+ (2,2′-azino-bis(3-ethylbenzothiazoline-6-sulfonic acid)), and NO• (nitric oxide), as detailed previously [8,49]. Generally, in 96-well flat-bottom microtitration plates, the samples were combined with radical solutions (DPPH•: 120 µM; ABTS•+: 7.4 mM; NO•: 10 mM) and left to incubate in darkness at room temperature for 30, 6 and 90 min, respectively. Butylated hydroxyanisole (BHA) was used as positive control for the DPPH• and ABTS•+, and ascorbic acid for the NO•. Absorbance measurements were taken at 517 nm for DPPH•, 734 nm for ABTS•+ and 546 for NO•.

The extracts’ ability to reduce Fe^3+^ was evaluated following the method outlined by Rodrigues et al. [8]. In 96-well plates, the samples were mixed with distilled water and 1% potassium ferricyanide, followed by incubation at 50 °C for 20 min. Subsequently, 10% trichloroacetic acid and 0.1% ferric chloride solution were added. An increase in absorbance at 700 nm indicated higher reducing activity, and the results were expressed as a percentage of inhibition relative to the positive control at a concentration of 1 mg/mL. BHA was used as a positive control for the FRAP assay.

Copper (CCA) and iron chelating activity (ICA), along with the positive control (EDTA), were evaluated in 96-well microplates following the procedures described by Rodrigues et al. [8]. For CCA, samples were combined with 50 mM Na acetate buffer (pH 6), 4 mM pyrocatechol violet, and a 50 µg/mL CuSO_4_ solution. For ICA, samples were mixed with distilled water and a 0.1 mg/mL FeCl_2_ solution. After 30 min, a 40 mM ferrozine solution was added, and the change in absorbance was measured at 632 nm for CCA and 562 nm for ICA, respectively.

#### 3.4.2. In Vitro Anti-Inflammatory Activity

The anti-inflammatory capacity of the extracts was evaluated stimulating RAW 264.7 macrophages to produce nitric oxide (NO) by means of lipopolysaccharide (LPS), following Rodrigues et al. [9]. The macrophage RAW 264.7 cell line was bought to CLS Cell Lines Service GmbH (Cytion catalog number 400319, Eppelheim, Germany). Cells were maintained in RPMI medium (supplementation: 10% heat-inactivated fetal bovine serum, 1% 2 mM L-glutamine, and 1% 50 U/mL penicillin/50 μg/mL streptomycin), at 37 °C in humidified atmosphere 5% CO_2_. Firstly, the MTT (3-(4,5-dimethylthiazol-2-yl)-2,5-diphenyltetrazolium bromide) method to assess cellular viability was employed to determine the concentration of the extracts that was non-cytotoxic to the cells. Cells were seeded at 10 × 10^3^ cells/well and left incubating for 24 h with extracts at 100, 50 and 25 µg/mL (diluted in culture medium). Cells incubated in culture medium with DMSO (in the same proportion as in the extracts) were used as a negative control. 

Once established, the concentration of the extracts that allowed more than 80% of cell viability (extracts at 25 µg/mL), extracts were incubated for 24 h with cells seeded at 2.5 × 10^5^ cells/well (medium serum- and phenol-free) and LPS (at 25 µg/mL). Cells incubated in culture medium with DMSO (same proportion as in the extracts) and LPS-stimulated were used as negative controls. Following the Griess method, using sodium nitrite as the standard in the calibration curve, the NO production was determined. Results were expressed as % of NO decrease relative to the control.

### 3.5. Statistical Analysis

Experiments were performed in triplicate and results were expressed as mean ± standard deviation (SD). To curve fit data and obtain EC_50_ values, GraphPad Prism 8.4.3 for Mac (GraphPad Software, Sand Diego, CA, USA) was used. Statistical differences (*p* < 0.05) were analyzed with XLSTAT trial version (Addinsoft 2023, New York, NY, USA) by one-way analysis of variance (ANOVA) and the post hoc Tukey multiple comparison test (assuming parametricity of the data). For the Principal Component Analysis, XLSTAT trial version (Addinsoft 2023, New York, NY, USA) was also employed. 

## 4. Conclusions

The investigation into the ecological and seasonal dynamics of *Limonium algarvense* Erben, commonly known as sea lavender, revealed significant influences on the synthesis of secondary metabolites and its biological properties. The biomass collected from distinct geographic locations, specifically “Ria de Alvor” in Portimão and “Ria Formosa” in Tavira, displayed notable variations in total phenolics and condensed tannins. Samples from “Ria de Alvor” during spring and autumn exhibited the highest content, accompanied by enhanced antioxidant and anti-inflammatory activities. Certain compounds exhibited season-specific and location-dependent variations. For example, during the summer, increased concentrations of luteolin, naringenin, and two apigenin derivatives were observed, whereas a myricetin derivative was more prevalent in the spring. Additionally, myricetin-3-*O*-rhamnoside and theasinensin B were more abundant in samples from “Ria Formosa”, while galactinol dihydrate was found in larger quantities in plants from “Ria de Alvor”. Seasonal changes were linked to environmental elements, notably temperature, whose excessive elevation can negatively impact plant metabolism. Furthermore, the presence of flowers and seeds in samples collected during spring and autumn appears to play a role in increasing the levels of polyphenols, consequently enhancing the biological properties of these samples. Additionally, differences observed among ecotypes (geographical locations) may be attributed to genetic factors. Overall, sea lavender plants from “Ria de Alvor” demonstrated favorable conditions for producing bioactive molecules, particularly during spring and autumn, correlating with the highest biological properties observed in these samples. These findings contribute valuable insights into the phytochemical changes and biological activities of *L. algarvense*, suggesting its potential as a source for natural products with health benefits.

## Figures and Tables

**Table 2:** Antioxidant activity (EC_50_ values, µg/mL) of *Limonium algarvense* (sea lavender) extracts prepared from biomass collected from two locations, in different seasons (Figure 2): radical scavenging on DPPH•, ABTS•+, and NO radicals, ferric reducing antioxidant power (FRAP) and metal-chelating activities on copper (CCA) and iron (ICA).

Location/Standards	Season	DPPH•	ABTS•^+^	NO	FRAP	CCA	ICA
(A) Ria de Alvor	Winter	125 ± 6 ^c^	26.9 ± 3.7 ^a^	498 ± 38 ^e^	298 ± 23 ^de^	272 ± 15 ^d^	Na
	Spring	45.6 ± 5.6 ^a^	73.7 ± 4.8 ^b^	315 ± 22 ^d^	333 ± 35 ^e^	176 ± 7 ^ab^	Na
	Summer	88.3 ± 8.1 ^b^	91.1 ± 5.0 ^bc^	703 ± 48 ^f^	240 ± 2. ^c^	338 ± 10 ^e^	Na
	Autumn	44.5 ± 4.1 ^a^	118 ± 4 ^d^	424 ± 72 ^e^	61.3 ± 1.1 ^a^	184 ± 8 ^b^	Na
(B) Ria Formosa	Winter	92.8 ± 12.0 ^bc^	19.6 ± 4.0 ^a^	68.0 ± 2.7 ^a^	173 ± 14 ^b^	231 ± 11 ^c^	Na
	Spring	62.7 ± 7.5 ^ab^	75.1 ± 4.7 ^b^	114 ± 10 ^ab^	273 ± 22 ^cd^	185 ± 4 ^b^	Na
	Summer	85.2 ± 2.4 ^b^	103 ± 2 ^cd^	270 ± 7 ^cd^	298 ± 11 ^de^	355 ± 14 ^e^	Na
	Autumn	65.6 ± 3.3 ^ab^	72.1 ± 7.6 ^b^	179 ± 12 ^bc^	63.3 ± 2.8 ^a^	193 ± 7 ^b^	Na
Positive controls	BHA	604 ± 31 ^d^	330 ± 23 ^e^		156 ± 7 ^b^		
	EDTA					156 ± 3 ^a^	28.4 ± 0.3
	Ascorbic acid			1713 ± 18 ^g^			

Na: non-active (activity lower than 50% up to 1000 µg/mL). Data represent the mean ± standard deview (SD) of at least three experiments performed in triplicate (*n* = 9). For each assay (column), different letters represent significant differences (*p* < 0.05).

**Table 3:** Meteorological values of mean temperatures (minimum and maximum) and total precipitation registered in the collection months (source: IPMA [52]).

Season	Month	$\bar{X}$ Min. Temp.	$\bar{X}$ Max. Temp.	Total Precipitation
Winter	January	8.9 °C	16.6 °C	29.6 mm
Spring	May	17.0 °C	24.7 °C	37.5 mm
Summer	July	21.6 °C	30.3 °C	0.0 mm
Autumn	November	13.7 °C	20.0 °C	155.8 mm

$\bar{X}$ min. temp.: mean of the minimum temperature; $\bar{X}$ max. temp.: mean of the maximum temperature.

## Data Availability

The data presented in this study are available on request from the corresponding author. The data are not publicly available due to proprietary restrictions imposed by our funding partners and collaborators.
